# Assessment of Activities of Daily Living and Quality of Life Among the Elderly in the Rural Area of Tiruvallur District, Tamil Nadu, India: A Cross-Sectional Study

**DOI:** 10.7759/cureus.80600

**Published:** 2025-03-15

**Authors:** Saranya Kumaran, Aishwarya P. M., Arun Raja, B. N. Surya

**Affiliations:** 1 Community Medicine, Sree Balaji Medical College and Hospital, Chennai, IND; 2 Preventive Medicine, Vinayaka Mission's Medical College and Hospital, Karaikal, IND; 3 Preventive Medicine, Chettinad Hospital and Research Institute, Chennai, IND

**Keywords:** adl, ageing, elderly, physical activity, qol

## Abstract

Introduction: The ageing population in India is growing faster than expected. With improvements in overall living standards and increased life expectancy, the quality of life (QOL) of the elderly needs greater attention. Assessing QOL reflects both the health status and overall well-being of elderly individuals. Additionally, the activities of daily living (ADL) play a crucial role in determining functional independence, as they assess an individual’s ability to perform essential tasks such as bathing, dressing, eating, and mobility. Evaluating both QOL and ADL helps in understanding the challenges faced by the elderly, enabling the development of targeted interventions for better health outcomes.

Materials and methods: A cross-sectional study was conducted to assess the QOL and performance of ADL among 250 elderly subjects visiting the Rural Health Training Centre (RHTC) of a private medical college in Chennai, Tamil Nadu. After obtaining consent from the study participants, interviews were conducted following ethical committee approval.

Results: The majority of the study participants (72%) were in the age group of 60-69 years, while 28% were above 70 years of age. Out of the 250 study participants, 155 (62%) were female and 95 (38%) were male. Among these, 42% were dependent on others for social and financial support. The overall mean scores of QOL of elderly people living in rural areas were found to be average, except for the mean score of social domain, which was very low. The mean scores for the environmental domain were higher compared to all other domains of QOL, indicating that elderly individuals living in rural areas were more satisfied with their environment. As age increases, dependence on performing daily activities also increases. However, physical independence was higher across different age and sex demographic variables, with a notable impact on activities under ADL.

Conclusion: The study found that elderly individuals in rural areas had a mean QOL score, with social relationships scoring the lowest. Dependence on daily activities increased with age, while physical activity showed a positive correlation with QOL. The findings emphasize the need for health education and community-based programs to promote functional independence and social engagement among the elderly.

## Introduction

India is ageing much faster than expected, and it is likely that by the year 2050, nearly 20% of its population will be 60 years and older [1]. The World Health Organization (WHO) reports show that there are more than 600 million elderly individuals worldwide, and this number is estimated to double by 2030 and reach two billion by 2050 [2]. Given the rapid rate of population ageing that developing countries like India are experiencing, there is a pressing need to focus on problems related to ageing and to plan corrective measures to improve the health status, well-being, and quality of life (QOL) of the elderly [3]. The living standards of the country’s population have improved, which in turn has increased the life expectancy of the elderly; however, the QOL for this demographic is still at risk [4].

QOL of the elderly encompasses their health status and overall well-being. As stated by WHO, QOL is defined as "an individual’s perception of their position in life with respect to the culture and values systems in which they live and in relation to their expectations, goals, concerns, and standards" [5]. QOL involves multiple dimensions, including social, psychological, physical, and economic components. An assessment of the QOL of the elderly broadly covers their needs and emphasizes the medical and psychological difficulties faced by geriatric populations [6]. Physical activity is an important determinant of QOL and has been positively linked with it [7,8]. Engaging in regular physical activity enhances psychological well-being, reduces functional decline, and promotes independence [9].

Research in this discipline is essential for understanding the QOL among the elderly. Existing studies suggest that the needs and challenges of older adults vary significantly based on socio-economic conditions, age, health status, and living arrangements [4]. However, there remains a need to explore how these factors collectively influence both QOL and activities of daily living (ADL). This study assessed both QOL and ADL of elderly individuals aged 60 years and above, and it also aimed to investigate the various factors associated with their QOL.

## Materials and methods

A cross-sectional study was conducted to assess the performance of ADL and QOL among the elderly in the rural area of Tiruvallur district. The study took place at the Rural Health Training Centre (RHTC). The study population included elderly subjects (age groups ≥60 years). Elderly patients who were unwilling or not in a position to provide information were excluded from the study. The research was conducted from March 2018 to May 2018, over a period of two months.

Sample size

The sample size was calculated based on a study conducted by Mahaur et al. [10], which reported a prevalence of 65% and an absolute precision of 6%. At a 95% confidence interval, the sample size was calculated to be 243. To ensure a whole number and to account for potential non-responses or data inconsistencies, the sample size was rounded up to 250. A systematic random sampling method was employed to select respondents at the RHTC. On average, 75 eligible patients visited the center daily. To ensure an unbiased and representative selection, every third patient was chosen for participation. This process was repeated daily until the required sample size of 250 respondents was achieved. If a selected patient did not meet the inclusion criteria or refused participation, the next eligible patient was considered.

Study tool

After obtaining written informed consent, a face-to-face interview was conducted with the participants. The World Health Organization Quality of Life-Brief Version (WHOQOL-BREF) questionnaire was used to assess the QOL of the elderly population. The questionnaire consists of four domains: physical, psychological, social relationships, and environmental health, containing 26 questions related to each domain [5]. A 5-point Likert scale was used to rate these domains. As per the WHO guidelines, 25 raw scores for each domain were calculated by summing the values of single items, which were then transformed into a score ranging from 0 to 100, where 100 represents the highest QOL and 0 the lowest. The mean score of each domain from the total score was calculated. The Katz Index of ADL was used to assess the functional independence of elderly individuals in performing daily activities, such as bathing, dressing, toileting, and mobility [6]. The WHOQOL-BREF questionnaire was available in a validated Tamil version; however, the Katz Index did not have a validated Tamil version. Data were collected using an interviewer-administered method, whereby questions were conveyed directly to participants by the interviewer.

Statistical analysis

Data collection, entry, and analysis were done using the Statistical Package for the Social Sciences (SPSS), Version 16.0 (SPSS Inc., Chicago, United States). Descriptive statistics were calculated for background variables, including socio-demographic characteristics. Findings for each domain were expressed in terms of mean and standard deviation. The differences between mean scores were tested using an independent sample t-test. To check the association between two categorical variables, a chi-square test was performed, and a correlation test was used to assess the linear relationship between QOL and ADL. A p-value of less than 0.05 was considered statistically significant.

## Results

Table 1 shows the socio-economic and living conditions of the elderly, which are crucial factors influencing their overall QOL and well-being. In this study, out of 250 participants, the majority, 180 (72%), belonged to the age group of 60-69 years, while 70 (28%) were aged above 70 years. Among the participants, 155 (62%) were female, and 105 (42%) had studied up to secondary education. In terms of marital status, 120 (48%) were widowed, highlighting a significant proportion of elderly individuals living without a spouse. Among the participants, 144 (58%) lived with their children, whereas 106 (42%) lived independently or with others. Additionally, 152 (61%) of the elderly were financially dependent, while 98 (39%) were financially independent, emphasizing the economic challenges faced by a considerable portion of the ageing population.

**Table 1:** Socio-demographic profile of the participants

Characteristics	Frequency (N=250)	Percentage (%)
Age (in years)		
60-69	180	72
≥70	70	28
Sex		
Female	155	62
Male	95	38
Education		
Illiterate	115	46
Primary education	30	12
Secondary education	105	42
Marital status		
Married	130	52
Widowed	120	48
Living with children		
Yes	144	58
No	106	42
Financial dependency		
Yes	152	61
No	98	39

Table 2 presents mean scores for physical, psychological, and environmental domains, which resulted in statistically significant associations in the context of age (p=0.009). There was no statistically significant association between QOL scores and the sex of the study participants. The physical domain score did not show a statistically significant association with marital status; however, other domains, such as environmental (p=0.019), psychological (p=0.007), and social (p=0.002), indicated statistically significant associations with marital status. The psychological (p=0.017) and environmental (p=0.045) domains also showed statistically significant associations with educational status. 

**Table 2:** Association of demographic variables with mean quality of life scores *p<0.05, statistically significant

Characteristics	Physical domain (mean ± SD)	Psychological domain (mean ± SD)	Social domain (mean ± SD)	Environmental domain (mean ± SD)
Age (in years)				
60-69 (n=180)	59.9 ± 15.7	62.03 ± 19.0	21.2 ± 12.02	66.5 ± 15.8
≥70 (n=70)	46.6 ± 14.3	46.4 ± 9.64	15.4 ± 13.4	53.1 ± 12.7
t value	6.22	7.51	3.31	6.42
p value	0.009*	<0.001*	0.143	0.007*
Sex				
Female (n=155)	56.1 ± 16.9	56.5 ± 17.4	18.2 ± 12.06	62.2 ± 17.4
Male (n=95)	56.3 ± 15.8	59.4 ± 19.9	21.9 ± 13.38	63.6 ± 14.2
t value	-0.09	-1.24	-2.27	-0.67
p value	0.953	0.598	0.324	0.777
Marital status				
Married (n=130)	59.4 ± 16.5	64.2 ± 19.4	24.6 ± 11.4	67.9 ± 15.6
Widowed (n=120)	52.6 ± 15.6	50.5 ± 14.0	14.2 ± 11.6	57.2 ± 15.1
t value	3.45	6.49	7.38	5.62
p value	0.143	0.007*	0.002*	0.019*
Educational status				
Illiterate (n=115)	51.5 ± 17.05	51.08 ± 16.2	16.3 ± 11.3	57.8 ± 16.5
Literate (n=135)	60.1 ± 14.8	63.2 ± 18.2	22.5 ± 13.6	67.0 ± 14.8
t value	-4.42	-5.72	-4.09	-4.82
p value	0.062	0.017*	0.081	0.045*

Table 3 presents the dependence of elderly across six components of daily activities: bathing, dressing, toileting, mobility, continence, and feeding, with dependence primarily observed among individuals aged 70 years and above.

**Table 3:** Activities of daily living among the elderly PD: physical dependency; PI: physical independency

Activities of daily living	Bathing		Dressing		Toilet use		Mobility		Continence		Feeding	
	PD, n (%)	PI, n (%)	PD, n (%)	PI, n (%)	PD, n (%)	PI, n (%)	PD, n (%)	PI, n (%)	PD, n (%)	PI, n (%)	PD, n (%)	PI, n (%)
Gender												
Female (n=155)	4 (2.6)	151 (97.4)	3 (1.9)	152 (98.1)	5 (3.2)	150 (96.8)	6 (3.9)	149 (96.1)	4 (2.6)	151 (97.4)	3 (1.9)	152 (98.1)
Male (n=95)	3 (3.2)	92 (96.8)	2 (2.1)	93 (97.9)	3 (3.2)	92 (96.8)	4 (4.2)	91 (95.8)	3 (3.2)	92 (96.8)	2 (2.1)	93 (97.9)
Age (in years)												
60-69 (n=180)	3 (1.7)	177 (98.3)	6 (3.3)	174 (96.7)	3 (1.7)	177 (98.3)	5 (2.8)	173 (97.2)	2 (1.1)	178 (98.9)	1 (0.6)	179 (99.4)
≥70 (n=70)	4 (5.7)	66 (94.3)	3 (4.3)	67 (95.7)	4 (5.7)	66 (94.3)	6 (8.6)	64 (91.4)	4 (5.7)	66 (94.3)	3 (4.3)	67 (95.7)

Across both genders, a higher percentage of elderly individuals maintained physical independence in all six activities. Among female patients, 151 (97.4%) were physically independent in bathing, 152 (98.1%) in dressing, 150 (96.8%) in toilet use, 149 (96.1%) in mobility, 151 (97.4%) in continence, and 152 (98.1%) in feeding. Similarly, male patients also displayed high levels of independence, with 92 (96.8%) independent in bathing, 93 (97.9%) in dressing, 92 (96.8%) in toilet use, 91 (95.8%) in mobility, 92 (96.8%) in continence, and 93 (97.9%) in feeding.

When analysed by age group, individuals aged 60-69 years demonstrated higher independence, with over 177 (98.3%) maintaining independence in bathing, toilet use, continence, and feeding. In contrast, elderly individuals aged 70 years and above showed slightly lower independence, with 66 (94.3%) independent in bathing, toilet use, and continence, and 64 (91.4%) in mobility, indicating a greater tendency for physical dependency in mobility-related activities. However, the overall trend suggested that a majority of the elderly population remains physically independent in performing daily activities.

Table 4 highlights demographic observations, showing that about 170 (94.4%) of the elderly aged 60-69 years displayed physical independence. Almost 55 (78.6%) participants aged 70 years or older also demonstrated physical independence in executing ADL. This indicated a potential for extreme statistical significance (p<0.001). However, in terms of sex demographics, there was statistical insignificance (p=0.193). Among the elderly, 143 (92.3%) female participants and 82 (86.3%) male participants exhibited a higher tendency toward physical independence.

**Table 4:** Association of demographic variables with activities of daily living *p<0.05, statistically significant PD: physical dependency; PI: physical independency If the participants required assistance in more than one activity, they were classified as physically dependent.

Characteristics	PD, n (%)	PI, n (%)	χ² value	p value
Age (in years)				
60-69 (n=180)	10 (5.6)	170 (94.4)	13.62	<0.001*
≥70 (n=70)	15 (21.4)	55 (78.6)		
Sex				
Female (n=155)	12 (7.7)	143 (92.3)	2.26	0.193
Male (n=95)	13 (13.7)	82 (86.3)		

Table 5 presents the correlation analysis between ADL and QOL among the elderly, revealing significant positive relationships across all domains. A strong correlation was observed between ADL and both physical (r=0.754, p<0.001) and environmental (r=0.712, p<0.001) QOL, indicating that elderly individuals with greater independence in daily activities tend to experience better physical health and environmental well-being. Additionally, a moderate correlation was found with social QOL (r=0.533, p<0.001), suggesting that higher functional independence is associated with improved social interactions and relationships. The psychological QOL domain showed a relatively weaker but still significant correlation (r=0.354, p<0.001), indicating that independence in daily activities contributes to better mental well-being.

**Table 5:** Correlation between activities of daily living and quality of life among the elderly *p<0.05, statistically significant

Quality of life	r	p value
Physical	0.754	<0.001*
Social	0.533	<0.001*
Psychological	0.354	<0.001*
Environmental	0.712	<0.001*

## Discussion

In this present study, the assessment of QOL across different domains revealed that the mean scores for the physical, psychological, and environmental domains were significantly associated with age (p-value <0.05). However, no significant association was found between QOL scores and gender. Marital status showed a significant association with the psychological, social, and environmental domains, but not with the physical domain. Educational status was significantly associated with the psychological and environmental domains. These associations are corroborated by the study conducted by George et al. [11], which reported similar determinants influencing the QOL among the rural elderly.

In terms of ADL, the present study found that physical dependency increased with age. Specifically, 10 (5.6%) individuals aged 60-69 years and 15 (21.4%) of those aged 70 years and above were physically dependent, indicating a significant association between age and physical dependency (p-value <0.001). Gender did not show a significant association with physical dependency. These findings are consistent with the research by Patel et al. [12], which highlighted age as a critical factor influencing ADL performance among the elderly in rural India.

The marital status analysis in this study showed that 130 (52%) participants were married, and 120 (48%) were widowed. The significant proportion of widowed individuals underscores the social challenges faced by the elderly, particularly women, in rural settings. This finding is supported by the study conducted by Dongre and Deshmukh [13], which found that widowhood adversely affects the QOL of the rural elderly.

Our study findings align with those of Eid et al. [14], who reported a significant positive correlation between ADL and QOL among older adults. Similar to their findings (r=0.280, p<0.001), our study demonstrated that greater independence in daily activities is associated with improved QOL, particularly in the physical (r=0.754, p<0.001) and environmental (r=0.712, p<0.001) domains. These results emphasize the importance of functional independence in promoting better health and well-being among the elderly. Additionally, both studies found a moderate correlation with social QOL, suggesting that maintaining ADL enhances social interactions and relationships.

Limitations

The major limitation of this study was that causal relationships between variables could not be established due to its cross-sectional design. Additionally, there was a possibility of self-reporting bias and recall bias among participants. To minimize self-reporting and recall bias, future studies can incorporate objective assessments and adopt a longitudinal approach for more reliable data collection. The study's external validity was limited, as it was confined to a single rural location; however, future research can improve generalizability by including participants from multiple geographic regions using random sampling. Further research is needed to establish causal relationships between elderly individuals and associated factors.

## Conclusions

This study highlights that age, gender, and physical dependency significantly impact the QOL and ADL among the elderly. The findings indicate that elderly individuals with greater physical dependency are at a higher risk of poor QOL, emphasizing the need for targeted interventions. Health education and preventive care programs should be implemented to improve physical and psychological health outcomes. Additionally, the government has a crucial role in addressing the needs of the elderly by developing and evaluating policies that enhance healthcare access, mobility support, and financial security. Community-based initiatives, such as peer support programs, can also play a vital role in fostering social stability among older adults. Future research should conduct an in-depth analysis of the factors affecting QOL and ADL while exploring longitudinal studies to better understand causal relationships. Addressing these aspects through a holistic and sustainable approach will contribute to an improved QOL for the elderly.
